# Pain in the Blood? Envisioning Mechanism-Based Diagnoses and Biomarkers in Clinical Pain Medicine

**DOI:** 10.3390/diagnostics5010084

**Published:** 2015-03-17

**Authors:** Emmanuel Bäckryd

**Affiliations:** 1 Division of Community Medicine, Department of Medical and Health Sciences, Faculty of Health Sciences, Linköping University, SE-581 85 Linköping, Sweden; 2 Pain and Rehabilitation Centre, Anaesthetics, Operations and Specialty Surgery Centre, Region Östergötland, SE-581 85 Linköping, Sweden; E-Mail: emmanuel.backryd@regionostergotland.se; Tel.: +46-10-103-3661

**Keywords:** biomarker, biopsychosocial, diagnosis, neuropathic, nociception, nociceptive, pain

## Abstract

Chronic pain is highly prevalent, and pain medicine lacks objective biomarkers to guide diagnosis and choice of treatment. The current U.S. “opioid epidemic” is a reminder of the paucity of effective and safe treatment options. Traditional pain diagnoses according to the International Classification of Diseases are often unspecific, and analgesics are often prescribed on a trial-and-error basis. In contrast to this current state of affairs, the vision of future mechanism-based diagnoses of chronic pain conditions is presented in this non-technical paper, focusing on the need for biomarkers and the theoretical complexity of the task. Pain is and will remain a subjective experience, and as such is not objectively measurable. Therefore, the concept of “noci-marker” is presented as an alternative to “pain biomarker”, the goal being to find objective, measurable correlates of the pathophysiological processes involved in different chronic pain conditions. This vision entails a call for more translational pain research in order to bridge the gap between clinical pain medicine and preclinical science.

## 1. Introduction

As exemplified by the deleterious consequences of congenital insensitivity to pain [1], acute pain is necessary for survival and has been called a “homeostatic emotion” [2]. Pain can persist long after tissue damage resolves, and chronic pain is usually defined as pain lasting more than 3–6 months [3]. It is sometimes stated that the vital importance of acute pain might be an important background to why pain can become chronic. According to this view, because the organism cannot afford missing potential life-threatening damage, the nervous system is heavily biased in favor of pain sensitivity [4].

According to the International Association for the Study of Pain (IASP), pain is an unpleasant sensory and emotional experience associated with actual or potential tissue damage, or described in terms of such damage. A basic conceptual difference is often made between acute, chronic, and cancer pain [3,5,6]. Although chronic pain itself is a very heterogeneous category, it has been called a “disease in its own right” [7]; the related concepts of pathological pain, and maladaptive pain, are important in this respect [8,9]. The prevalence of chronic pain is estimated to be as high as 20%, generating high costs both at the individual and societal level [3,10]. Against this background, it is not surprising that chronic pain has been described as a public health issue [11]. At the same time, the so-called opioid epidemic in the US [12,13] is a reminder of the paucity of effective, safe, and evidence-based treatment options for chronic pain.

The aim of this non-technical paper is to introduce the reader to the vision of mechanism-based diagnoses of chronic pain conditions, focusing on the need for biomarkers and the theoretical complexity of the task.

## 2. What is a Biomarker?

A biomarker has been defined as a characteristic that is objectively measured and evaluated as an indicator of normal biological processes, pathogenic processes, or pharmacologic responses to a therapeutic intervention [14]. Hence, biomarkers can help in diagnosis, prognosis, the evaluation of treatment response, the development of drugs; they can also serve as surrogate endpoints, *i.e.*, as substitutes for clinical endpoints [15,16]. Biomarkers can also be of value in helping to predict which individuals are at risk for a certain event [17], e.g., the development of chronic post-operative pain. Objective biomarkers that could complement the subjective assessment of pain would therefore be a big step forward for pain medicine [15].

It is important to acknowledge that the concept of biomarker can include many different kinds of measurements in addition to the analysis of substances in body fluids, e.g., sensory phenotyping, intraepidermal nerve fiber counts, microneurography, electrophysiological recording of noxious stimulus-evoked cortical potentials, different kinds of functional imaging, or experimental pain tests (such as pressure-pain thresholds or conditioned pain modulation) [17,18,19,20,21,22,23,24].

## 3. Pain Biomarkers: A Contradiction in Terms?

As pain by definition is a subjective experience (see the IASP definition above), it has been contended that finding biomarkers for pain is a sheer impossibility [25]. According to this view, there is a logical contradiction (and an ethical danger) in the attempts to find objective biomarkers for subjective states like pain. In the context of this debate, I have elsewhere proposed that the neologism “noci-marker” would perhaps be a better term than “pain biomarker” for denoting attempts to find objective, measurable correlates to the neurobiological processes involved in different pain conditions [26,27]. This proposal pertains to the now classical philosophical distinction between the “hard” *vs.* “easy” problems of consciousness [28], and also to the well-known distinction between nociception and pain (nociception being the neural process of encoding noxious stimuli). Pain is always subjective and cannot be observed “from the outside”; it is a lived reality, an “inner” experience. In contrast, the neural circuits and the biochemistry of the nociceptive pathways can be studied “from the outside” by science.

It is important to underline that the prefix “noci” in “noci-marker” does not refer to the well-known distinction between nociceptive and neuropathic (or other types of) pain (for a definition of these terms, see Section 4). Rather, the prefix “noci” does, in this context, refer to the broader concept of there being a nociceptive system in the human body [29]. Researchers can study this system “from the outside”, just like every other physiological system. And the nociceptive system can, like all other systems, be afflicted by pathologies—hence the concept of pathological pain, when pain becomes a “disease in its own right” (e.g., neuropathic pain) [7,8,9]. For example, we have elsewhere proposed that low levels of beta-endorphin in the cerebrospinal fluid might be a noci-marker (albeit probably not a very specific one) of refractory postoperative/posttraumatic neuropathic pain [27].

In the pain setting, biomarkers should not be seen as a substitute to the patient’s subjective report. A long-term vision for pain medicine could be the possibility of basing the prescription of analgesics (or even disease-modifying drugs) on a mechanistic understanding of different pain types. Such a vision requires the discovery of mechanism-specific biomarkers [30]. Today, analgesics are often prescribed on a trial-and-error basis.

## 4. What is a Chronic Pain Diagnosis?

The complexity of pain taxonomy is illustrated by how the classification system issued by IASP is constructed [31]. This classification system has five axes: region of the body; organ system involved; temporal characteristics; intensity and time since onset; etiology. The resulting five-digit code is too complicated to be used in clinical practice, but it illustrates the complexity and heterogeneity of pain conditions. In clinical practice, pain diagnoses according to the International Classification of Diseases (ICD-10) are often based on anatomical location. For instance, unspecific “low back pain” (ICD-10 code M54.5) is a very frequent but (from a pathophysiological point of view) obviously not very useful pain diagnosis. In contrast to this kind of unspecific diagnoses, the need for mechanism-based pain diagnoses has been underlined [30].

The contrast between nociceptive and neuropathic pain is one of few mechanism-based distinctions in pain medicine. In contrast to nociceptive pain, which is caused by activation of peripheral nociceptors (due to actual or potential tissue damage) [32], neuropathic pain is due to a disease or lesion in the somatosensory nervous system itself [33]. Although this distinction is therapeutically important, it is also essential to recognize that both nociceptive and neuropathic pain are very heterogeneous categories. Inflammatory pain is often seen as a subtype of nociceptive pain [32] (nociceptors being sensitized by the process of inflammation), but some authors view inflammatory pain as a distinct subtype (see, e.g., Wolf [30]; in that case, nociceptive pain is defined as an early warning device that aims at protecting the organism from tissue damage.)

Future mechanism-based classifications will probably build on but also transcend the simple dichotomy between nociceptive and neuropathic pain. For instance, a group of pain physicians at the Swedish Quality Registry for Pain Rehabilitation has recently proposed that chronic widespread pain (generalized pain) should be viewed as a specific pain type, with a postulated (presently to a large degree unknown) core pathophysiological mechanism [34]. When the pain is caused by a severe psychiatric disease, it is called psychogenic (this should be clearly differentiated from the psychological co-morbidities commonly associated with chronic pain) [34]. Sometimes, the mechanism underlying the pain of an individual patient remains idiopathic. Hence, five pain types emerge: nociceptive (including inflammatory), neuropathic, widespread, psychogenic, and idiopathic.

The concept of central sensitization is often put forward in this context. The pioneering work of Clifford J. Woolf [35] led to the concept of central sensitization. Until the 1980s, pain processing was largely seen to work much like a telephone wire [36]. Today central sensitization, defined as a nociception-driven amplification of neural signaling within the central nervous system leading to pain hypersensitivity, is generally acknowledged to be of physiological importance in chronic pain conditions [36]. Clinically, central sensitization is inferred indirectly from allodynia (pain due to a stimulus that does not normally provoke pain) or hyperalgesia (increased pain from a stimulus that normally provokes pain).

Synaptic plasticity, described as long term potentiation (LTP), is an important general neurobiological principle underlying learning and memory [37]. In the pain setting, LTP is best seen as a particular component of central sensitization [36,38]. Moreover, central sensitization differs from the older concept of windup in that the former entails an amplification that outlasts the end of the conditioning stimuli, whereas the latter represents an increasing output during the course of a train of identical stimuli [36]. Windup does not in itself have any long-term consequences, but in conjunction with central sensitization it can greatly enhance the nociceptive output of the dorsal horn [38]. Moreover, central sensitization has been divided into an acute and a late phase. The acute phase is dependent on nociceptor input to the spinal cord (activity dependent), whereas the late phase entails transcriptional changes (transcription dependent) [30].

The term central sensitization seems to be increasingly used in clinical pain medicine [39], and it has been invoked in a wide range of different pain conditions: complex regional pain syndrome, fibromyalgia, miscellaneous musculoskeletal disorders, neuropathic pain, osteoarthritis, post-surgical pain, rheumatoid arthritis, temporomandibular disorders, tension-type headache, visceral pain hypersensitivity syndromes [36]. Given the broadness of the concept, it has been claimed that central sensitization should probably not be viewed as a specific pain type [34]. Moreover, it is important to remember that it is a neurophysiological term which can only be applied when both neural input and output are known [39]. Hence, strictly speaking, the concept of central sensitization should be restricted to the preclinical neurophysiological setting. Nonetheless, some authors use concepts like “central sensitivity syndromes” or “centralized pain” to characterize some of the clinical pain conditions listed above in this paragraph [40,41]. Despite these on-going discussions about whether the term should be used in clinical pain medicine or not, there is a broad consensus in the pain community that central sensitization is an essential concept to grasp and have in mind when treating pain patients. The concept is also a powerful pedagogical tool, as it helps frustrated patients getting a sense of being understood. Central sensitization makes pain hypersensitivity “real”.

## 5. The Biopsychosocial Model

Research into the neurobiology of pain mechanisms is essential, but it is often stated that pain is a biopsychosocial phenomenon [42]. According to the biopsychosocial model, presented by Engel in 1977 [43,44], biological, psychological, and social factors interact in an intricate and indissoluble manner. This model has had a great impact on pain medicine [42]. Engel argued that the prevailing biomedical model, with its reductive physicalism and mind-body dualism, had come to a dead end. Engel was not denying the overwhelming advances of modern medicine, but he contended that there was a need for a more holistic reframing of medical science. In his own words, the doctor “must weigh the relative contributions of social and psychological as well as of biological factors implicated in the patient’s dysphoria and dysfunction as well as in his decision to accept or not accept patienthood and with it the responsibility to cooperate in his own health care”.

Pain is not equivalent to nociception. Nociception is the neural process of encoding noxious stimuli; pain is a subjective experience. This experience is often described as having three different aspects: a sensory-discriminative aspect, an affective-motivational aspect, and a cognitive-evaluative aspect [45]. The biopsychosocial model fits well with such a multifaceted view of pain. For instance, it is widely recognized that affective factors like fear [46] and depression [47] are important to assess in pain patients, although the causal relationships are complex and difficult to analyze [47,48]. The biopsychosocial model is consistent with the view of the brain as an active system that filters, selects and, thanks to descending neural pathways, modulates nociceptive input from the periphery [41,45,49,50]. Hence, the human experience of pain is best viewed as a complex, holistic multi-level reality consisting of biological processes, psychological experiences and socio-cultural contexts.

A substantial part of the knowledge about pain mechanisms has been acquired through animal experiments, and when pondering the biopsychosocial model of pain, it is wise to remember that humans and animals are different. Although there are obvious similarities between species, there are also differences. This is not least the case in such a multi-facetted experience as pain, and translating evidence from animals to humans in this field is far from trivial [51]. The failure of Neurokinin-1 receptor antagonists (Substance P antagonists) in humans, despite encouraging preclinical findings, is a classic example of this [52]. Another aspect of translatability is the need to take age, gender, and ethnicity into consideration. Here, translatability has bearing on the concept of personalized medicine, and biomarkers are crucial in this respect [53].

Against the background of what has been described in Section 3, Section 4 and Section 5, it is believed the reader will appreciate the theoretical complexity and the challenges inherent in trying to find biomarkers for different pain conditions.

## 6. Biomarkers in Pain Medicine: What is the State of the Play?

While acknowledging the importance of a broad biopsychosocial perspective on pain, and while recognizing the theoretical difficulties described in Section 3, Section 4 and Section 5, I contend that the endeavor to find noci-markers is far from meaningless. It is, of course, a daunting task, and Borsook *et al.* [15] have very well listed many of its challenges: pain intensity scales lack objectivity; pain is a complex experience; adaptive changes can occur over time; there may be confounding effects due pharmacological interventions or placebo; there may be multiple mechanisms at work; tolerance or other long-term effects of analgesics may cloud the picture.

A long-term vision for pain medicine could be the possibility of basing the prescription of analgesics on a mechanistic understanding of different pain types, using a panel of “noci-markers” as diagnostic aide [30]. Today, analgesics are often prescribed on a trial-and-error basis. This is in sharp contrast to, e.g., cardiology, where chemical biomarkers play a role well-known to all physicians. Instead of today’s focus on symptom relief, a better understanding and assessment of different pain mechanisms would perhaps even enable clinicians and researchers to develop disease-modifying drugs for chronic pain. The move from merely symptom control to disease modification is arguably an appealing vision for pain medicine.

The cerebrospinal fluid (CSF) is an interesting target for human biomarker studies in neurological disorders, including pain (e.g., Alzheimer’s disease, multiple sclerosis, or pathological pain conditions) [54,55,56,57]. In 2003, Mannes *et al.* [58] described Cystatin C in the CSF as a biomarker of acute labor pain in humans. A year later, a larger study failed to reproduce these findings [59]. The rise and fall of Cystatin C as a potential biomarker of acute pain can be described as a paradigmatic example of a (failed?) “candidate-protein approach” [60]: based on, e.g., animal experimental data, a specific protein is hypothesized as being a potential biomarker (a “candidate protein”), and a comparative study in humans is then carried out in order to test the hypothesis. Other candidate proteins in earlier studies have included neurotrophic factors [61,62], cytokines [62], and neuropeptides such as nociceptin [63,64], beta-endorphin [27,65,66], or substance P [67,68,69].

Although there are some good examples of single protein markers used in clinical medicine, it has been said that a single protein biomarker in many cases is unlikely to clearly discriminate between a specific disease and healthy controls; a more viable approach is to look at the combination pattern of several proteins [70]. Hence, in contrast to the traditional “candidate protein approach”, the use of high throughput proteomic analytic techniques may be a possible way forward [71], perhaps combined with modern multivariate data analysis [72,73,74,75,76]. (The proteome is the total protein content in a specific cell, body fluid or tissue. Whereas the genome is constant, the proteome is constantly modulated by genome-environment interactions [77].) Hence, successful noci-marker studies in the future will probably require a combination of laboratory and bioinformatics skills. The new systems biology tool Pain Networks should be mentioned in that respect [78].

Of course, taking a blood sample is much less invasive than performing a lumbar puncture, and blood biomarkers are therefore more appealing than CSF biomarkers. This paper being part of the special issue “biomarkers in blood”, I will now briefly review some blood biomarker studies in the field of pain medicine, focusing on (1) neuropathic pain; (2) osteoarthritis; and (3) fibromyalgia.

In patients with chronic neuropathic pain, our group failed to detect differences in plasma levels of neuropeptides Beta-endorphin and Substance P [27]. In contrast, studies investigating systemic low-level inflammation (defined as 2–4 fold elevations in circulating levels of cytokines [79]), seem to indicate that neuroinflammation associated with the chemokine-cytokine network as consequence of nerve damage probably plays an important role in the pathogenesis of neuropathic pain [80]. In the following, I will briefly focus on IL-6. A recent study of 110 patients with lumbar radicular pain showed that high serum IL-6 levels were associated with less favourable recovery in patients with lumbar radicular pain [81]. Patients with neuropathic pain, due to either herniated intervertebral disc or carpal tunnel syndrome, had significantly higher plasma levels of IL-6 (and TNF) compared to healthy controls [82]. Patients who developed post-herpetic neuralgia after herpes zoster had significantly higher levels of IL-6 in serum than those with herpes zoster who did not develop neuralgia; both groups had higher levels of IL-6, IL-1β, TNF, and IL-8 than controls; and pain severity in neuralgia correlated positively with IL-6 levels [83]. However, the literature is not univocal concerning IL-6 [84,85,86]. Nonetheless, the balance of evidence suggests that IL-6 functions as an algesic mediator following nerve injury, not least because it activates spinal cord microglia [87].

A study of patients with painful osteoarthritis has also reported increases in plasma IL-6 [62]. Looking at potential blood biomarkers for painful osteoarthritis, namely three collagen markers and two inflammation markers (high-sensitivity C-reactive protein and matrix metalloproteinase-mediated breakdown of C-reactive protein), Arendt-Nilsen *et al.* [24], in a methodologically interesting study, recently reported the associations between these five biochemical markers and experimental markers of pain (pressure–pain thresholds, temporal summation, and conditioned pain modulation). In another recent study on low back pain patients, other potential blood biomarkers were investigated [88]. Hence, biomarkers for osteoarthritis are being investigated by many groups.

Blood cytokines are of interest as markers of fibromyalgia. Concerning IL-6, fibromyalgia syndrome patients have been reported to have increased levels of this cytokine in blood [89]. Other potential biomarkers have been investigated in fibromyalgia, e.g., the neuropeptide Substance P. Although levels of Substance P in the CSF have been shown to be high in fibromyalgia [68,69], blood levels have not differed [90]. However, the biomarker technique that has been most studied in fibromyalgia is arguably the Conditioned Pain Modulation test (CPM). CPM measures the Diffuse Noxious Inhibitory Controls (DNIC) system [91], and deficient DNIC (*i.e.*, deficient top-down inhibition of pain) has been predominantly demonstrated in fibromyalgia [17]. However, many pain conditions are thought to be (at least in part) caused by abnormal top-down control of pain [41], and CPM is used widely in pain research.

## 7. Conclusions

Given that the field of biomarkers for pain conditions (“noci-markers”) is still in its infancy, the idea of basing the choice of analgesics or even disease-modifying drugs on a blood test may seem more like science fiction than science. Still, this vision for the future of pain medicine is appealing for pain clinicians: what if in the future we were able to prescribe analgesics or disease-modifying drugs not on a trial-and-error basis, but on the basis of information given by a blood test? Once again, it has to be underlined that this would not be a measurement of the chronic pain experience itself. Rather, it would be an objective measurement of the underlying pathophysiological mechanisms. Hence, the vision of future “noci-markers” in pain medicine is not antithetical to a broad biopsychosocial view of pain, and it does not entail a rejection of the IASP definition of pain.

We will of course never be able to detect the pain experience itself in the blood—there can be no biomarkers for pain itself. However, given the blood-brain barrier, is it even conceivable that one could detect pathophysiological changes in the central nervous system via a panel of noci-markers in the blood? This is an important question that presupposes that the blood-brain barrier is unaffected in chronic pain conditions. Do we know that this is the case? Be that as it may, if the vision of finding noci-markers in the blood would turn out to be an impossibility, the CSF would remain available as a possible target. For those of us who are pain clinicians, the vision described in this article is an incentive for more translational research into the mechanisms of chronic pain. The task is immense. So is the need.
